# Supplementary material for: Type 2 Diabetes Prediction Model in China: A Five-Year Systematic Review
Source: Healthcare (Basel). 2025 Aug 15;13(16):2007. doi: 10.3390/healthcare13162007 (PMC12385379; doi:10.3390/healthcare13162007)
Supplement: Supplementary file 1 [file healthcare-13-02007-s001.zip › Supplementary file S1.pdf]

China National Knowledge Infrastructure (CNKI,中国知网)
#1: 2型糖尿病 (type 2 diabetes) OR 二型糖尿病 (type 2 diabetes) OR T2D OR T2DM OR 非胰岛素依赖型糖尿病 (non-insulin-dependent diabetes) OR 成人发病型糖尿病 (adult-onset diabetes) OR 成熟发病型糖尿病 (maturity-onset diabetes) OR NIDDM OR 成年型糖尿病 (adult-type diabetes) OR 成人糖尿病 (adult diabetes)
#2: 风险预测 (risk prediction) OR 预测模型 (prediction model) OR 风险模型 (risk model) OR 风险评估 (risk assessment) OR 风险评分 (risk score) OR 危险评分(hazard score) OR 预测工具 (prediction tool) OR 预后模型 (prognostic model) OR 多因素预测 (multivariable prediction) OR 列线图 (nomogram) OR nomogram OR logistic 回归 (logistic regression) OR Cox 回归 (Cox regression) OR ROC 曲线 (ROC curve) OR 校准 (calibration) OR 判别 (discrimination) OR AUC OR c-statistic
#3: 中国 (China) OR 中国人 (Chinese) OR 中华人民共和国 (People's Republic of China) OR PRC OR 中国大陆 (Mainland China) OR Mainland China OR 华东 (East China) OR 华南 (South China) OR 华北 (North China) OR 西南 (Southwest China) OR 西北 (Northwest China)
#4: #1 AND #2 AND #3
Hits: 229

PubMed
#1: "Diabetes Mellitus, Type 2"[MeSH Terms] OR "Type 2 Diabetes"[Title/Abstract] OR "Type II Diabetes"[Title/Abstract] OR T2D[Title/Abstract] OR T2DM[Title/Abstract] OR "Non–Insulin-Dependent Diabetes Mellitus"[Title/Abstract] OR NIDDM[Title/Abstract] OR "Adult-Onset Diabetes"[Title/Abstract] OR "Maturity-Onset Diabetes"[Title/Abstract] OR "Ketosis-Resistant Diabetes"[Title/Abstract] OR "Ketosis-Prone Diabetes"[Title/Abstract]
#2: "risk prediction"[Title/Abstract] OR "predictive model"[Title/Abstract] OR "prediction model"[Title/Abstract] OR "risk model"[Title/Abstract] OR "risk assessment"[Title/Abstract] OR "prognostic model"[Title/Abstract] OR nomogram[Title/Abstract] OR "risk score"[Title/Abstract] OR "risk stratification"[Title/Abstract] OR "c-statistic"[Title/Abstract] OR AUC[Title/Abstract] OR ROC[Title/Abstract] OR calibration[Title/Abstract] OR discrimination[Title/Abstract] OR predict*[Title/Abstract] OR model*[Title/Abstract]
#3: China[Title/Abstract] OR Chinese[Title/Abstract] OR "People's Republic of China"[Title/Abstract] OR PRC[Title/Abstract] OR "Mainland China"[Title/Abstract] OR "Chinese population"[Title/Abstract]
#4: #1 AND #2 AND #3
Hits: 416

Web of Science
#1 TS=("Type 2 Diabetes Mellitus" OR "Type II Diabetes" OR T2D OR T2DM OR NIDDM OR "Non-Insulin-Dependent Diabetes Mellitus" OR "Adult-Onset Diabetes" OR "Maturity-Onset Diabetes" OR "Ketosis-Resistant Diabetes")
#2 TS=("risk prediction" OR "prediction model" OR "predictive model" OR "risk mo

del" OR "risk assessment" OR "prognostic model" OR nomogram OR "risk score" OR "risk stratification" OR c-statistic OR AUC OR ROC OR calibration OR discrimination OR predict* OR model*)
#3 TS=(China OR Chinese OR PRC OR "People's Republic of China" OR "Mainland China" OR "Chinese population")
#4 #1 AND #2 AND #3
Hits: 435

Table S1. Exclusion criteria and quantitative statistics

Exclusion category	Exclusion criteria	Reasons for exclusion	Number of excluded articles
Data integrity issues	Missing key diagnostic information, incomplete treatment records, or obvious errors/omissions in data collection	Insufficient data reduces the validity of the analysis and the reliability of the conclusions and may lead to bias	26
Non-Chinese population	The study population is not located in China, or the target population is not the general adult population in mainland China.	Sample baseline characteristics do not match the Chinese population and cannot be generalized to our core study target (Chinese general adults)	67
Specific high-risk groups	Recruit only high-risk subgroups such as obesity and hypertension (e.g., pregnant women, children, mentally challenged)	Studies of specific subgroups do not reflect the risk of T2D in the general population, reducing the external validity of the conclusions.	44
Disputes over data sources	Unstandardized data sources (e.g., manual recording of data leading to large errors), doubtful quality of sources	Source data quality varies, directly affecting the reproducibility and scientific validity of the study	7
Multi-disease composite model	Endpoint events include composite risk prediction of T2D with other chronic diseases (e.g., cardiovascular, renal, etc.)	The model does not focus on the risk of a single incidence of T2D, and the output does not allow for the interpretation of T2D risk in isolation	29
T2D Complications Risk Model	Predictive models focusing on diabetic complications (e.g., retinopathy, nephropathy, neuropathy, etc.)	Prediction of complications is a different goal than the one explored in this paper, and the purpose of the study is incompatible	113
Research process integrity issues	Incomplete documentation of experimental processes, lack of adequate follow-up information or failure to obtain ethical approval	Inability to assess the quality of research design and implementation, reducing the credibility of findings	31
Molecular/cellular/genetic level research	Basic research at the molecular biological or genetic level (cell line, genome, proteome, etc.)	Focus on mechanism exploration, not predictive models constructed with clinical or population epidemiology data	25
